# α-Synuclein conformers reveal link to clinical heterogeneity of α-synucleinopathies

**DOI:** 10.1186/s40035-023-00342-4

**Published:** 2023-03-14

**Authors:** Matthias Schmitz, Niccolò Candelise, Sezgi Canaslan, Hermann C. Altmeppen, Jakob Matschke, Markus Glatzel, Neelam Younas, Saima Zafar, Peter Hermann, Inga Zerr

**Affiliations:** 1 Department of Neurology, National Reference Center for TSE, The German Center for Neurodegenerative Diseases (DZNE), Georg-August-University, University Medicine Gottingen, Goettingen, Germany; 2 National Center for Drug Research and Evaluation, Institute Superiore di Sanità, Rome, Italy; 3 Institute of Neuropathology, University Medical Center Hamburg-Eppendorf (UKE), Hamburg, Germany

**Keywords:** α-synucleinopathies, α-synuclein, RT-QuIC, Protein strains

## Abstract

α-Synucleinopathies, such as Parkinson’s disease (PD), dementia with Lewy bodies (DLB) and multiple system atrophy, are a class of neurodegenerative diseases exhibiting intracellular inclusions of misfolded α-synuclein (αSyn), referred to as Lewy bodies or oligodendroglial cytoplasmic inclusions (Papp–Lantos bodies). Even though the specific cellular distribution of aggregated αSyn differs in PD and DLB patients, both groups show a significant pathological overlap, raising the discussion of whether PD and DLB are the same or different diseases. Besides clinical investigation, we will focus in addition on methodologies, such as protein seeding assays (real-time quaking-induced conversion), to discriminate between different types of α-synucleinopathies. This approach relies on the seeding conversion properties of misfolded αSyn, supporting the hypothesis that different conformers of misfolded αSyn may occur in different types of α-synucleinopathies. Understanding the pathological processes influencing the disease progression and phenotype, provoked by different αSyn conformers, will be important for a personalized medical treatment in future.

## Introduction

The characteristic hallmark of synucleinopathies is the presence of misfolded α-synuclein (αSyn) in the form of intra-neuronal aggregates called Lewy bodies (LBs) and Lewy neurites (LNs) [1]. The most common dementias [2], besides AD, are dementia with Lewy bodies (DLB), Parkinson’s disease (PD) and PD-associated dementia (PD-D), accounting for 1%–2% of the total population aged 65 years or over [3]. Whereas DLB and PD-D are frequently considered as opposing edges of the same pathological spectrum [3–5], distinctions have been described [3], supporting the hypothesis that they are different diseases.

Here, we will review the similarities and differences between both types of α-synucleinopathies. We will first discuss their clinical and neuropathological features and summarize current knowledge on the spreading of these diseases. In this regard, we will outline the prion-like spreading behaviour of misfolded αSyn, suggesting the occurrence of different αSyn conformers. Those disease-specific conformers of the misfolded αSyn may play a role in the pathogenesis of the disease and explain different syndromes. The symptoms of PD and DLB can overlap and rapid eye movement (REM) sleep behaviour disorder (RBD) can be associated with PD, DLB, and multiple system atrophy (MSA), making it difficult to differentiate between these conditions based on clinical presentation alone. Misfolded αSyn may play a role in the development of PD, DLB, and MSA, and different conformers of αSyn may be involved in their pathogenesis. Researchers are working to understand the mechanisms behind the misfolding and spreading of αSyn and to identify potential therapeutic targets. PD, DLB, and MSA are typically diagnosed through a combination of clinical assessment, imaging, and biomarker analysis.

The role of alternative pathogenic conformations of misfolded proteins, defined as structural polymorphisms or strains, as well as the selective vulnerability of the neuronal populations to different conformers, will be addressed as a putative mechanism behind the phenotypic variability of these diseases. We will recapitulate the improvements in the methodologies aimed at the discrimination among α-synucleinopathies with potential diagnostic applications.

Moreover, we focus on the recent progress in the application of protein seeding assays for the diagnosis of α-synucleinopathies. Although not yet included in the diagnostic criteria, recent evidence has highlighted the high diagnostic accuracy of misfolded αSyn detection, pointing toward a possible inclusion in future revisions of the diagnostic criteria for these pathologies.

## Comparison of α-synucleinopathies: are PD and DLB the same or different diseases?

### Clinical features of α-synucleinopathies

Parkinsonism (tremor, rigidity, and bradykinesia) is a clinical feature of α-synucleinopathies (PD, DLB, and MSA) and many other neurological diseases. Most differential diagnoses, such as MSA [6], tauopathies (progressive supranuclear palsy and corticobasal degeneration) [7], and vascular parkinsonism [8] usually show distinct clinical syndromes. Nonetheless, inaccurate clinical diagnosis of PD may be a common phenomenon, especially in non-specialized centres [9].

In the frame of parkinsonism, DLB and PD-D share neuropathological features of αSyn and LB as well as a variation of clinical features, including cognitive impairments and hallucination [10]. The DLB diagnosis is based on general consensus criteria when cognitive symptoms precede motor pathology by 1 year at least, whereas PD-D is diagnosed when motor impairment occurs at least 1 year before cognitive deficits [11]. Clinical features include the onset of hallucinations, which typically occur spontaneously in DLB, possibly related to temporal lobe dysfunctions, whereas in PD-D, they often appear after dopaminergic treatment [12]. Rigidity and bradykinesia are common parkinsonian features in DLB, whilst tremors at rest are less common. RBD is commonly associated with DLB and should precede cognitive impairment by several years [13]. Besides RBD, hyposmia, constipation and depression are considered to be prodromal symptoms that could help in the differential diagnosis of PD-D versus DLB [13–15]. DLB is a type of dementia that is characterized by a more rapid decline in cognitive abilities such as executive function, episodic memory, attention, and constructive abilities compared to PD-D (another type of dementia) [3]. These differences are more evident during early and mid-stages of the pathologies. Nevertheless, they tend to converge at later stages of the disease [16]. Magnetic resonance imaging studies showed greater grey matter loss in parietal, occipital and frontotemporal areas in DLB, which correlate with the visual-spatial hallucination [15]. The selective impairment of neurotransmitter systems also differs between DLB and PD-D. Indeed, whereas the striatal dopaminergic system [17] and the cholinergic system [18] in the basal forebrain and pedunculopontine nucleus are impaired in PD-D, the cortical serotoninergic systems are more impaired in DLB compared to PD [19]. Furthermore, higher tau pathology and amyloid-β deposition have been found in DLB compared to PD-D [20–23]. To sum up, clinical differences between PD-D and DLB are probably related to disease stage and the sequence of affected anatomic, biochemical or functional compounds of the CNS. In principle, the two diseases may completely mimic each other at some points of the clinical course.

**Fig. 1:**
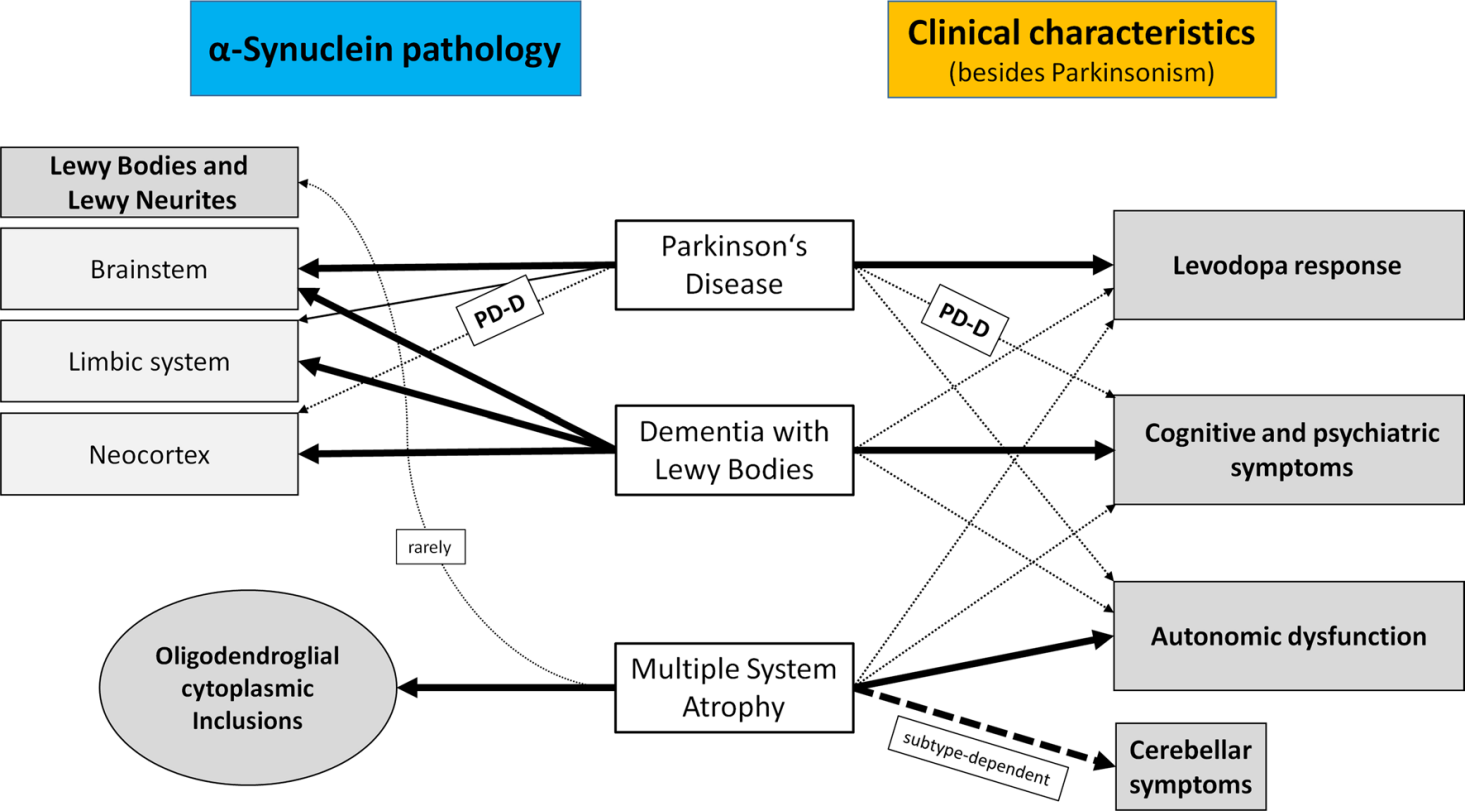
Clinical characteristics and neuropathological findings in PD, DLB, and MSA. The figure displays important clinical characteristics (right side) as well as neuropathological findings (left side) of the three most important forms of α-synucleinopathy. Bold black arrows point to typical aspects, whereas thin arrows point to neuropathological and clinical findings that are either less frequent or only occur in late disease stages. *PD-D* Parkinson’s disease-associated dementia

**Fig. 2:**
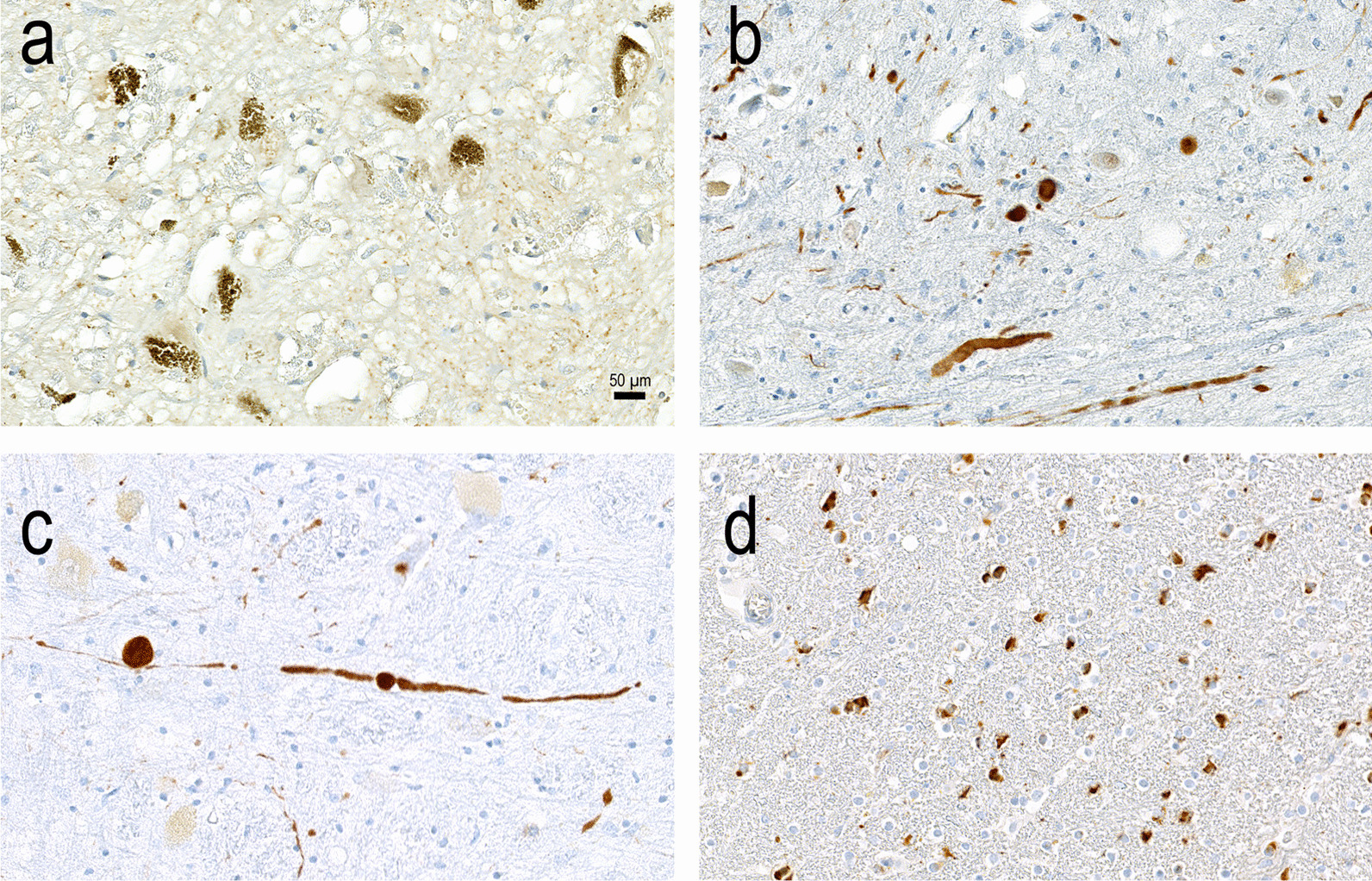
Immunohistochemical detection of α-synuclein (αSyn) deposits in different types of α-synucleinopathies. Original immunohistochemical staining from 2-µm sections of the cerebral cortex, cerebral white matter, and the midbrain including the substantia nigra was done on a Ventana Benchmark XT Autostainer (Ventana, Tucson, AZ), in accordance with the manufacturer's recommendations, using antibodies against αSyn (polyclonal; Zytomed 519–2684; dilution 1:1000). **a** Negative control. A person with no clinical history of Parkinson’s disease (PD), PD-associated dementia or dementia with Lewy bodies (DLB) showed physiological neuromelanin-containing neurons in the substantia nigra but no αSyn-positive structures. **b** A patient with a history of PD showed numerous αSyn-positive Lewy bodies and neurites in the substantia nigra. **c** αSyn-positive Lewy neurites in the neocortex of a patient with DLB are shown. **d** Numerous αSyn-positive glial cytoplasmic inclusions are recognizable in the white matter of a patient with multiple system atrophy

Although the sum of clinical manifestations and the neurobiological changes may indicate differences among synucleinopathies, there is a high degree of overlap not only among synucleinopathies, but also among other neurodegenerative diseases that may appear as parkinsonism but do not show overt αSyn pathology, such as Alzheimer’s disease, corticobasal degeneration and progressive supranuclear palsy [24] (Fig. 1). Therefore, the definitive diagnosis still relies on the post-mortem observation of neuropathological changes associated with the presence of LBs and LNs throughout the nervous system. Detection of LBs and oligodendroglia cytoplasmic inclusions (GCIs) in MSA can be achieved by immunohistochemical staining (Fig. 2a–d). While LBs and GCIs are distinguishable, and LBs in PD and DLB may be observed in different brain regions, they are not discriminable from appearance and structure in the brain tissue (Fig. 2b–d). Control persons without αSyn pathology contain no LBs or GCI; they show neuromelanin-containing cells in the substantia nigra instead (Fig. 2a).

### Staging of α-synucleinopathies and different subtypes

α-Synucleinopathies can be divided into different stages, recapitulating the disease progression useful for sample characterization [25, 26]. According to the LB distribution and the progression of pathology, DLB patients can be grouped according to their main pathologies in brainstem, limbic and neocortical. Lewy pathology can be assessed by different types of αSyn antibody [27], which is a major advantage allowing a better neuropathological characterization compared to previously used methods that rely on haematoxylin–eosin or ubiquitin staining [28]. A staging system for αSyn pathology in sporadic PD has been reported by Braak et al. [29]. It is useful to predict the routes of neuronal damage according to the selective susceptibility of neurons and to define the disease progression. According to Braak et al*.,* LB pathology originates from outside the CNS and follows a retrograde pathway through the vagus nerve, because αSyn aggregates had been found also in the peripheral autonomic nervous system [29–31], supporting the body-first model (pathogenic αSyn originating from the enteric nervous system and not from the CNS) [32]. Since the risk of developing dementia in the DLB has been associated with body-first symptoms, whereas brain-first symptoms are associated with PD, which shows a slower progression towards dementia [32], the spreading pathway is supposed to have an influence on the type of -α-synucleinopathy.

Braak stage I is defined by the presence of αSyn inclusions in the dorsal IX/X motor nucleus in the medulla oblongata, in the anterior olfactory nucleus as well as in the intermediate reticular zone. The LNs are found in a higher number than LBs. Braak stage II is characterized by the location of LBs and LNs in the reticular formation and in the caudal raphe nuclei. LBs and LNs are also found at early Braak stages (I–II) in incidental DLB cases (iDLB) in the substantia nigra, with no relationship between αSyn burden and neuronal loss [33]. Braak stage III is characterized by lesions in the magnocellular nuclei of the basal forebrain and in the substantia nigra, while stage IV by evident degeneration and depigmentation of the pars compacta of the substantia nigra. LNs can be detected in the amygdala and in the hippocampus, while LBs are detectable in the anteromedial temporal mesocortex. In Braak stage V, the LB pathology spreads into the neocortex, whilst the burden of lesions increases in the brainstem, olfactory bulb and substantia nigra. The advanced stage VI exhibits LBs and LNs throughout the whole neocortex.

Even though prodromal symptoms such as autonomic dysfunction can be identified by neuropathological diagnostic, the Braak stage classification is restricted to PD, and not applicable to DLB or amygdala-predominant Lewy pathology [34].

A similar classification system for LB pathology has been put forward by the DLB consortium for Lewy pathology, in which PD-D and DLB are grouped together [35, 36], including amygdala-dominant cases. The observation of αSyn types or strains exhibiting different features of spreading and influencing the susceptibility of neuronal subtypes, has been proposed to explain differences among α-synucleinopathies.

## Protein misfolding amplification assays for misfolded αSyn

The invention of amyloid seeding assays for the diagnosis of protein misfolding diseases has prompted research to develop faster and more accurate techniques. In this frame, a fundamental innovation, firstly in the prion field and later also for other prion-like diseases, was the development of the protein misfolding cyclic amplification (PMCA) [37]. This PMCA assay consists of cycles of incubation and sonication, causing the seeded conversion of monomers into pathogenic species and the breakage of longer fibrils into more reactive oligomers, respectively. The detection of the end-products of the reaction relies on Western blot after proteinase K (PK) digestion. To automatize the protein misfolding process and to reduce the assay duration, a new generation of protein misfolding amplification assay, based on shaking and application of a recombinant substrate has been developed. A major advancement of this assay is the substitution of sonication, which was reported to be difficult to control [38, 39].

This assay may produce a comparable output in a more standardized environment and, strikingly, result (depending on the type of substrate) in a probably non-infectious end-product in cerebrospinal fluid (CSF)-seeded reactions [40]. This novel assay, termed quaking-induced conversion (QuIC), was further refined by the addition of thioflavin-T (ThT), allowing real-time monitoring of the aggregation process. These advancements led to the development of the real-time quaking-induced conversion (RT-QuIC), firstly applied for prion diseases [41].

Later, the RT-QuIC and the PMCA protocols were modified and applied as very promising methods to diagnose α-synucleinopathies through detection of misfolded αSyn. Misfolded αSyn, which is prone to aggregation, may act as a seed for the template-induced conversion process of monomeric αSyn substrate [42–45].

The seeding-conversion process of αSyn aggregation allows the detection of minuscule amounts of misfolded seed. The αSyn RT-QuIC seeding response curve has a sigmoidal shape, showing a lag phase (small oligomers are included in a growing aggregate); afterwards, the fibrils become elongated until the aggregation process reaches a plateau when the supply of recombinant substrate is used up [42]. Fairfoul et al. analysed for the first time the template-induced seeding conversion of αSyn from body fluids (e.g., CSF) and tissue (e.g., brain) [43] and obtained an accurate discrimination of DLB and PD from controls (sensitivity 95% and specificity 92%–100%). A second study from Groveman et al. [46] reported a diagnostic performance of 100% specificity and 93% sensitivity to diagnose DLB and PD (Table 1).

**Table 1:** Overview of the diagnostic accuracy of the αSyn RT-QuIC assay in different studies

Reference	Source	Substrate	Accuracy indicated by sensitivity and specificity
Fairfoul et al. [43]	CSF	Hu FL αSyn	92%–95% sensitivity and 100% specificity for DLB and PD versus AD and controls; DLB (*n* = 12), PD (*n* = 22), AD (*n* = 30), mixed DLB/AD (*n* = 17), HC (*n* = 20), PD control (*n* = 15)
Saijo et al. [47]	CSF	His-WT, K23Q αSyn	92% sensitivity and 100% specificity for PD and DLB
Groveman et al. [46]	CSF	K23Q αSyn	93% sensitivity and 100% specificity; DLB (*n* = 17), PD (*n* = 12), AD (*n* = 16), non-α-synucleinopathy controls (*n* = 31)
Bongianni et al. [48]	CSF	Hu FL αSyn	92.9% sensitivity and 95.9% specificity synucleinopathies versus non‐α-synucleinopathy; αSyn (*n* = 27), non-αSyn (*n* = 49)
Garrido et al. [49]	CSF	Hu FL αSyn	40% LRRK2‐PD and 18.8% LRRK2‐NMC with positive RT-QuIC results; IPD with 90% sensitivity and 80% specificity LRRK2-PD (*n* = 15), LRRK2-NMC (*n* = 16), IPD (*n* = 10), HC (*n* = 10)
Manne et al. [44]	CSF	Hu FL αSyn	100% sensitivity and specificity; PD (*n* = 15) compared with controls (*n* = 11)
van Rumund et al. [50]	CSF	Hu FL αSyn	75% sensitivity and 85%–98% specificity; αSyn (*n* = 85), non-αSyn (*n* = 26)
Rossi et al. [51]	CSF	Hu FL αSyn	98% specificity and 95.3% sensitivity for α-synucleinopathies (DLB + PD + iRBD + PAF); LB-αSyn + (*n* = 21), LB-αSyn − (*n* = 101)
Shahnawaz et al. [52]	CSF	Hu FL αSyn	95.4% sensitivity to discriminate PD and MSA; *n* = 153
Orrú et al. [53]	CSF	K23Q αSyn	97% sensitivity and 87% specificity for PD versus controls PD (*n* = 108), HC (*n* = 85)
Bargar et al. [54]	CSF	Hu FL αSyn	98% sensitivity and 100% specificity PD (*n* = 88), DLB (*n* = 58), Controls (*n* = 68)
Rossi et al. [55]	CSF	Hu FL αSyn	95% sensitivity and 97% specificity for MCI-LB patients versus controls
Donadio et al. [56]	CSF	Hu FL αSyn	78% sensitivity and 100% specificity for α-synucleinopathies versus controls; α-synucleinopathies (*n* = 9), non-α-synucleinopathies (*n* = 24), controls (*n* = 16)
Manne et al. [57]	Skin	Hu FL αSyn	PD versus control: 96% sensitivity and 96% specificity for frozen skin tissues (PD *n* = 25, control *n* = 25); 75% sensitivity and 83% specificity for formalin-fixed paraffin-embedded skin sections (PD *n* = 12, control *n* = 12)
Wang et al., 2020 [58]	Skin	Hu FL αSyn	93% sensitivity and 93% specificity for synucleinopathies (PD, DLB, MSA) versus controls (AD, PSP, CBD, NNCs.); α-synucleinopathies (*n* = 57), controls (*n* = 73)
Mammana et al. [59]	Skin	K23Q αSyn	89.2% sensitivity and 96.3% specificity for DLB versus neurological controls; vitam (*n* = 69), post-mortem (*n* = 49)
Donadio et al. [56]	Skin	Hu FL αSyn	86% sensitivity and 80% specificity; α-synucleinopathies (*n* = 31), non-α-synucleinopathies (*n* = 38), controls (*n* = 24)
Stefani et al. [60]	OM	Hu FL αSyn	45.2% sensitivity and 89.9% specificity for RBD plus PD versus controls
Manne et al. [61]	SMG	Hu FL αSyn	100% sensitivity and 94% specificity; PD (*n* = 13), ILBD (*n* = 3), controls (*n* = 16)

*CSF* Cerebrospinal fluid, *OM* Olfactory mucosa, *SMG* Submandibular gland, *Hu FL* Human full-length

Manne and colleagues [44] observed αSyn-seeded aggregation in RT-QuIC reactions seeded with CSF when sodium dodecyl sulphate and zirconia/silica beads (which could promote aggregation of αSyn during the RT-QuIC assay) were added, and they reported a comparable performance to that by Fairfoul et al.

Candelise et al. established a pre-analytical fractionation protocol (removal of macromolecules and avoidance of artificial inducers) to isolate oligomeric and insoluble αSyn from brain homogenates, which allowed discrimination of DLB from PD according to the seeding conversion ability and efficiency of the isolated αSyn seed, supporting the hypothesis of different oligomeric αSyn conformers having different seeding conversion characteristics [45]. Analysis of the RT-QuIC products from DLB patients revealed formation of a fibrillary, PK-resistant αSyn. The selective detection of seeding activity in DLB was explained by the avoidance of artificial inducers (e.g., beads) and the pre-analytical conditions. Garrido and colleagues used an already published protocol [43, 49, 50, 62, 63] to detect αSyn aggregates in the CSF from PD; they obtained a sensitivity of 90% and a specificity of 80% in 10 PD cases [49].

Other studies detecting CSF αSyn via RT-QuIC have confirmed in larger cohorts the accuracy of CSF RT-QuIC for α-synucleinopathy diagnosis [51, 56] (Table 1). Besides the confirmation of high diagnostic accuracy towards PD and DLB, researchers have reported the ability of RT-QuIC to monitor αSyn aggregation using CSF from patients with idiopathic REM behaviour sleep disorder (iRDB) and pure autonomic failure (PAF) with a sensitivity above 90% [51]. Interestingly, iRBD and PAF are considered to be prodromal for the onset of DLB and PD, respectively [63, 64], hinting at the possibility of detecting these diseases in early stages, long before the motor or cognitive deficits appear.

## Discrimination of different αSyn strains

### Prion-like spreading characteristics of αSyn conformers

The spreading of αSyn inclusions is supposed to proceed in the caudo-rostral direction through neuronal transfer [65]. Evidence of neuronal spreading of αSyn pathology was first reported in patients with neuronal graft transplant, who developed Lewy pathology years after the surgical procedure [66–68] akin to the prion-like transmission [69]. Misfolded αSyn may either be actively released [70] by neurons through exocytosis [71] or following their death, spread to neighbouring neurons, where it is internalized following the endocytic pathway [72]. Passive diffusion and transfer through tunnelling nanotubes have been proposed as well [73, 74]. Spreading along neurites has been shown in different cell models. For instance, in mouse embryonic cultures, fibrillary αSyn was found to be transported in an anterograde direction and taken up by neighbour neurons [75]. Studies in rats showed the neuron-to-neuron spreading of pathogenic αSyn from the olfactory bulb toward non-olfactory areas [76]. Upon injection of pathogenic αSyn in the medulla oblongata of rats, caudal-to-rostral spreading of αSyn was observed, supporting the spreading along interconnected neurons [65]. A similar work was conducted by administering LBs from PD to non-human primates, which later developed PD pathology [77].

Together, these lines of evidence corroborate the central role of αSyn spreading in disease development and progression. Like most neurodegenerative diseases [78, 79], synucleinopathies are being recognized as protein-misfolding diseases [80], defined by the template-induced conversion process of one or more proteins. As such, αSyn has been shown with different structures which may lead to different clinical pictures and therefore different diseases. In the next section, we will focus on the impact that different conformations of αSyn may have on the phenotypic variability observed in synucleinopathies.

### Selective vulnerability towards misfolded αSyn

The sequence of occurrence of symptoms significantly differs between PD and DLB [34], which may reflect differences in the route of spreading of αSyn between the two pathologies [15]. Similarly, neuronal loss diverges between diseases as well as among neuronal areas and subpopulations involved (e.g., glutamatergic neurons in the hippocampus and dentate gyrus [81]). As neuronal populations are widely different, it is suggestive to speculate that each group of neurons displays a specific vulnerability towards different conformations of the same pathogenic seed. Selective vulnerability would account for the different brain regions affected in similar synucleinopathies, also supporting the differential spreading and staging of the diseases. Specifically, cortical neurons may be more sensitive to a DLB-derived strain, whereas substantia nigra areas may be more affected by a PD-derived seed, as reflected by the prevalence of cognitive symptoms observed in DLB compared to the prevalence of motor disorders found in PD. In a recently published paper, Candelise et al. brought evidence of a different seeding activity between PD and DLB (discussed in detail in the next section). They assayed both frontal cortex (FCx)- and substantia nigra pars compacta (SNc)-derived samples and compared the results from different brain regions of the same disease (PD or DLB) by two-way ANOVA [45], before and after the RT-QuIC aggregation assay. The analysis of the total content of αSyn before RT-QuIC showed no significant differences among brain regions of the same pathology. However, the seeding amplification  assay showed a significant increase of the signal derived from FCx of DLB patients when compared to reactions seeded with the substantia nigra of the same disease. This result indicates that FCx-derived seeds could promote αSyn aggregation more effciently compared to SNc-derived seeds, supporting the selective vulnerability of different brain areas toward αSyn. FCx neurons, mainly glutamatergic and GABAergic neurons, could be more sensitive toward DLB strains of αSyn, whereas cholinergic and dopaminergic SNc neurons would be less affected by the same αSyn strain, but still be capable of seeding the aggregation assay. This hypothesis is in line with DLB clinical manifestation, showing cortical function impairment prior to the motor symptoms dependent on the nigrostriatal pathway.

Proteinaceous strains are not only critical for the understanding of pathology spreading and the selective routes affected in various forms of synucleinopathies, but may also serve as a biomarker for early and selective detection of the pathology. They are emerging as a powerful diagnostic tool, due to the aggregation techniques that have been developed in the last decade. To this end, promising results have recently been put forward on the ability of aggregation assays to detect prodromal forms of synucleinopathies such as iRBD and PAF [51, 63]. Likewise, the recent successful amplification of αSyn seeds derived from skin biopsies of synucleinopathy patients [54, 56–58, 82], from saliva [83] and from olfactory swab [84] may offer the possibility of an accurate, ante-mortem diagnosis of synucleinopathies before the onset of symptoms.

### Evidence for the existence of different αSyn strains in α-synucleinopathies

Different folding conformations of pathogenic seeds (referred to as strains) have already been suggested to explain the clinical heterogeneity of neurodegenerative diseases. Different strains of a particular misfolded protein possess distinct biochemical and pathological conformational properties, which are maintained after serial transmissions [85, 86]. Previous studies reported a strain-like behaviour of αSyn regarding the transmissibility after inoculations in rodents [87–89]. This observation could explain the heterogenic phenotypes in α-synucleinopathies, based on the differential vulnerability of neuronal subset of brain regions toward conformers of the same protein. The prion-like behaviour of αSyn has been reported for MSA αSyn strain [88]. Evidence for the passage of αSyn aggregates from graft to host further corroborates the prion-like nature of αSyn [66–68]. The misfolded species may fold in different conformations and each of which may possess its own spreading characteristics  [89, 90].

A possibility to discriminate between different types of misfolded αSyn in α-synucleinopathies is the analysis of the seeding conversion behaviour of a misfolded αSyn seed either by RT-QuIC or by PMCA.

Candelise et al. recently showed that brain-derived fractions from PD and DLB patients subjected as seeds for RT-QuIC assay, exhibit different signal responses in the RT-QuIC assay [45]. Although DLB and PD are considered variants of the same disease spectrum [2, 3, 10], a positive RT-QuIC reaction was only obtained from DLB cases but not from PD, suggesting either the existence of different types of αSyn seeds in DLB and PD brains [45] or differential environmental factors influencing seeding conversion activity of a misfolded seed [42]. Although Western blot analysis did not show difference in the total amounts of isolated αSyn seeds between groups, the different intensities of seeding activity might also be affected by minuscule higher amounts of pathogenic αSyn seeds in the DLB cohort compared to PD.

In this context, it has been reported that PD-seeded reactions may show positive seeding responses as demonstrated by several groups [43, 47] employing other protocols, indicating the presence of seeding-competent αSyn in PD-derived material (Table 1).

The observed αSyn aggregation from two control cases that later on developed PD, strongly suggests the possibility of applying this detection system to prodromal stages of the pathology. Positive RT-QuIC results from prodromal cases of synucleinopathies, such as iRBD or PAF, have recently been reported in two studies involving large patient cohorts [51], further corroborating the ability of αSyn-seeded RT-QuIC to detect an early stage of the pathological aggregation.

Recently, van der Perren and co-workers reported differences in PMCA-amplified seeds derived from PD, DLB and MSA brain homogenates [91]. Interestingly, MSA strains show similarities with PD strains. However, MSA strains are more potent in provoking motoric deficits, nigrostriatal neurodegeneration, αSyn spreading, and inflammation, indicating a more aggressive nature of these strains. In contrast, DLB strains display more modest neuropathological features in experimental transmission experiments [91].

**Fig. 3:**
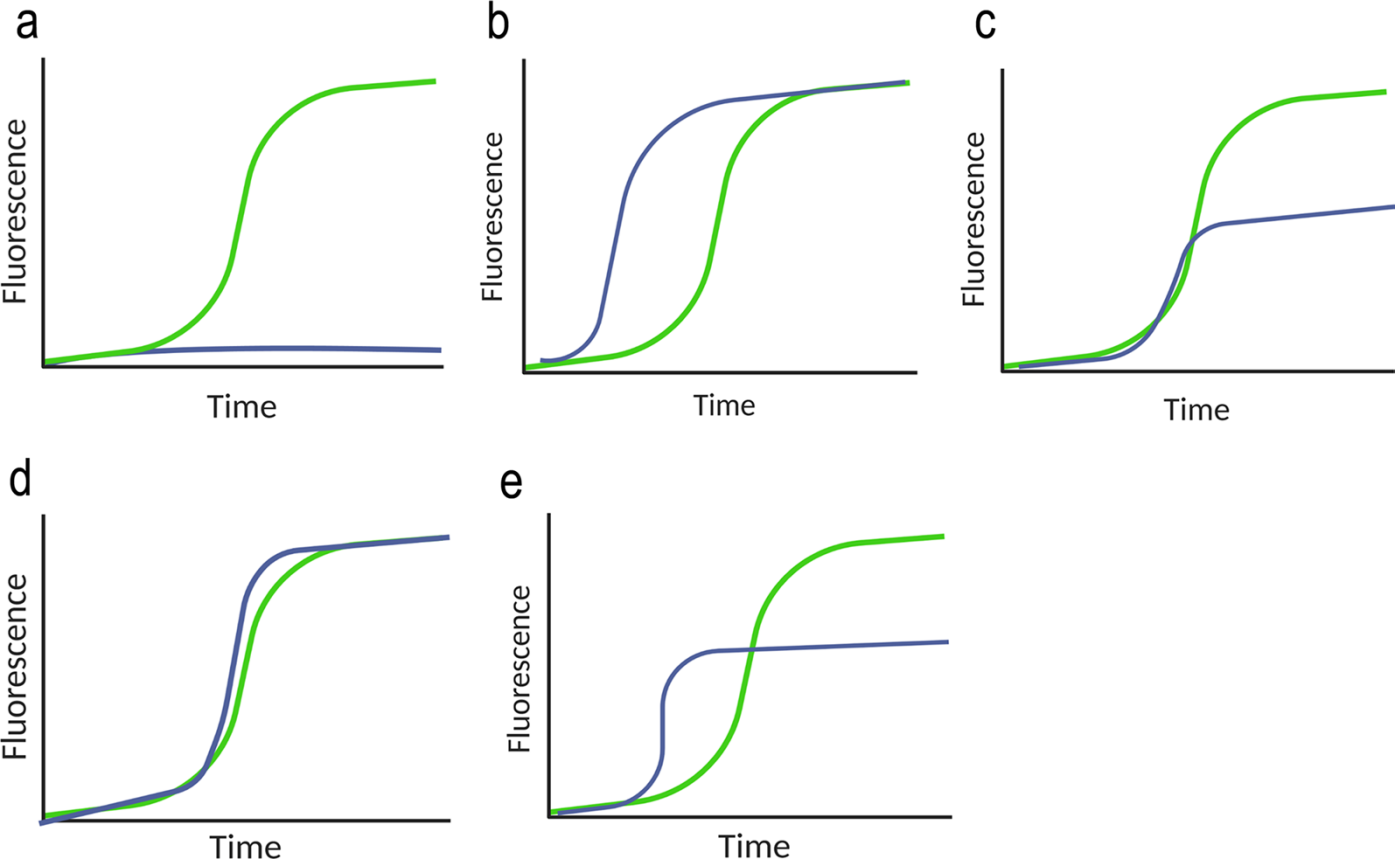
Potential strain typing by RT-QuIC. A schematic illustration indicating the possible outcomes of RT-QuIC to distinguish a classical signal (in blue) from various conditions. All-or-none response (**a)**, different lag-phases with the same I_Max_ (**b**), different I_Max_ with the same lag-phase (**c**), same lag-phase and I_Max_, but with steeper aggregation phase (different areas under the curve) (**d**), and different lag-phases and I_Max_ (**e**), can be applied to distinguish between different seeding efficiencies

Semi-quantitative parameters of the RT-QuIC may indicate potential strain distinctions, as different conformations of the same pathogenic seed could show differences in promoting the aggregation. A potential strain-typing scheme for the RT-QuIC response curve may apply semi-quantitative seeding parameters as published before [41]. For example, there are five theoretical RT-QuIC outcomes that may help in the discrimination of the strains. In the first scenario, the presence of signal indicates the ongoing conversion from a pathogenic seed scenario (Fig. 3a). This is the case of a case–control study, in which the material derived from the healthy patient is compared to a diseased one or when the signal could be detected only in one specific group of α-synucleinopathies (e.g., in DLB but not in PD), indicating a putative strain difference underlying the two pathologies [45].

If two pathogenic seeds are compared, various results could be observed. A seed may be faster in triggering the aggregation of its substrate, resulting in a shorter lag-phase (Fig. 3b). A similar result could also be obtained by the same seed applied at different concentrations, highlighting the importance of quantification of the seeds. Although having similar maximum intensities, the two putative strains may be distinguished both by their lag-phase and by area under the curve. In a different case (Fig. 3c), the lag-phases may be identical but the strain may be identified by the lower maximum of fluorescence and hence a lower area under the curve. This may reflect a lower ability of a seed to bind the substrate, resulting in a weaker aggregation. This may be the case observed in Groveman’s work [46], in which PD-seeded reactions showed a reduced maximal fluorescence signal during the run (I_Max_) compared to DLB-seeded reactions. This result may also be obtained by a strain with a less avid binding to the detecting dye, as reported by Ferreira and co-workers [92]. The most complex scenario (Fig. 3d) involves seeds with similar latency and avidity for the substrate (lag-phase and maximum intensity, respectively) but different exponential phases. A steeper curve would indicate a stronger ability to convert the substrate once the reaction is triggered. Here, although the maximum intensity and the lag-phases are similar, the two strains may be discriminated by the area under the curve values, with the steeper signal resulting in a larger area. Lastly, a seed may promote conversion faster than another but displaying a lower I_Max_ (Fig. 3e). This case resembles the observation reported by Shahnawaz and co-workers [52], with MSA-derived seeds showing a faster but less intense ThT-based PMCA signal compared to PD-derived seeds.

## Conclusion

The definitive ante-mortem diagnosis of α-synucleinopathies is a challenging topic of utmost importance, as parkinsonian symptoms are overlapping among similar neurodegenerative diseases. These pathologies may thus be misdiagnosed, leading to useless therapeutic interventions [93]. Different pathologies may arise from the same αSyn protein, whose spatial conformation may lead to different courses of disease affecting different brain regions. The optimization of protein seeding assays for the detection of misfolded αSyn in biological fluids leads to an encouraging increase in the ante-mortem diagnostic accuracy and the ability to discriminate among different αSyn strains, even detecting prodromal stages of the pathology. The type of αSyn conformer may influence the spreading of misfolded αSyn and, hence, the clinical manifestation of the pathology. Therefore, the diagnostic application of a fast and reliable protein seeding assay, such as the RT-QuIC, may provide useful information for the development of personalized (αSyn strain-specific) therapies aimed at inhibiting or even stopping the neurodegenerative processes in future.

## Data Availability

Not aplicable.

## Abbreviations

DLB: Dementia with Lewy bodies
LB: Lewy body
LN: Lewy neurite
αSyn: α-synuclein
MSA: Multiple system atrophy
PD: Parkinson’s disease
PD-D: PD-associated dementia
RT-QuIC: Real-time quaking-induced conversion
